# Editorial: New insights in pediatric gastrointestinal food allergies

**DOI:** 10.3389/falgy.2024.1531494

**Published:** 2024-12-11

**Authors:** Laura Carucci, Neil Shah, Roberto Berni Canani

**Affiliations:** ^1^ Department of Translational Medical Science, University of Naples “Federico II”, Naples, Italy; ^2^ ImmunoNutritionLab, CEINGE Advanced Biotechnologies Research Center, University of Naples “Federico II”, Naples, Italy; ^3^ Department of Pediatrics, Portland Hospital, London, United Kingdom; ^4^ Reckitt Healthcare, Slough, United Kingdom; ^5^ Task Force on Microbiome Studies, University of Naples “Federico II”, Naples, Italy; ^6^ NutriTechLab, University of Naples “Federico II”, Naples, Italy

**Keywords:** gut microbiome, cow milk protein allergy, ultraprocessed foods, advanced glycation end products, eosinophilic esophagitis, first 1000 days, short chain fatty acids

**Editorial on the Research Topic**
New insights in pediatric gastrointestinal food allergies

Pediatric food allergies (FA) are a significant and growing health concern, especially in developed countries. The most recent data on FA epidemiology in children, depicts a dramatic picture in terms of prevalence, incidence, and severity of FA (1, 2). Food allergy derives from a negative interaction between genetic background and environmental factors, leading to a breakdown of immune tolerance to food antigens (Carucci et al.).

During the last decades one of the most impressive changes in environmental factors was related to the increased exposure to the ultraprocessed foods (UPF), as underlined in the paper by Carucci et al. included in this issue. In Western countries, the consumption of UPF has increased at alarming rate, so that more than half of the total daily dietary energy intake in children derives from UPF exposure (Carucci et al., 3). The ready to use foods, their palatability, their low costs as well as their eye-catching appearance, make the UPF very attractive. At the same time UPF have a very poor nutritional profile, being highly processed, rich in sugar, fat and low in fibers (Carucci et al., 4).

The paper by Carucci et al. highlights the mechanistic role of environmental factors, especially UPF, in pathogenesis of FA and eosinophilic esophagitis (EoE). In this paper the authors underline how a specific component of UPF (i.e., the advanced glycation end products) activating the alarmin signal cause alteration of epithelial barrier with subsequent abnormal allergen exposure and activation of type 2 (Th2) inflammation. The result of this pathway may have a direct role to “switch on” EoE and FA inflammation, as also demonstrated by clinical and preclinical data (Carucci et al., 4, 5). These data could be relevant for defining innovative strategies for preventing and managing FA and EoE.

## Immune mechanisms

The interesting review by Nuyttens et al. elucidate the pathophysiology of gastrointestinal FA classifying in IgE-, non-IgE-mediated, and mixed forms, based on their immune mechanisms (Nuyttens et al.). Despite the presence/absence of specific IgE against foods, the immune tolerance failure and gut microbiome (GM) alterations are the mainstay of FA pathogenesis (7).

The pivotal role of GM and immune system in FA as led to the concept of “GM-immune system axis” (8). This mutual and dynamic interaction starts in early life, at the beginning of the first 1,000 days, a crucial time frame in which the axis is extremely vulnerable. During this period several environmental factors could negatively or positively influence the axis, setting the basis for current and future health status of the child (Carucci et al., 9).

**Figure 1:**
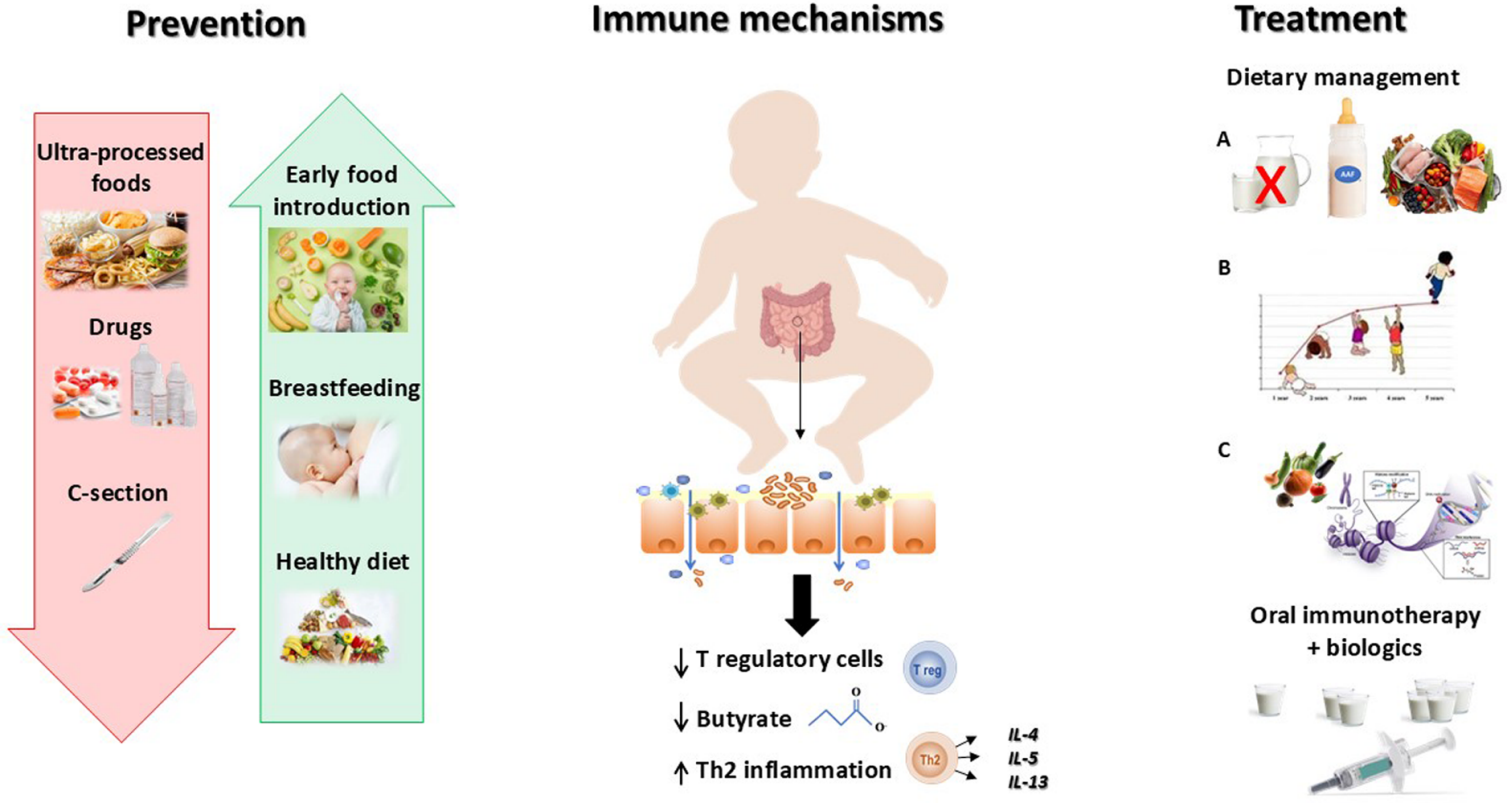
Better knowledge on pathogenesis is inspiring effective strategies against food allergy. Food allergy derives from a breakdown of immune tolerance due to a negative interaction between genetics and environmental factors. The development of the immune system is deeply regulated by the gut microbiome. An alteration of the gut barrier leading to the abnormal exposure to food allergens, induces a Th2 inflammatory response and a downregulation of T regulatory cells. Several environmental factors may act as trigger factors inducing gut barrier impairment, like ultraprocessed foods (UPF) consumption, antiseptic agents, and early drugs administration, as well as other factors reducing the biodiversity of the infant gut microbiome, including C—section. Based on this evidence it is important to reduce the exposure to all factors negatively impacting the gut-microbiome immune system axis (red arrow) and to increase the exposure to beneficial factors able to positively influence the axis (green arrows). Once developed, food allergy needs to be managed with a modern multistep approach including Dietary treatment that encompasses **(A)** Dietary education (allergen avoidance, food substitutes, and healthy diet), **(B)** Adequate intake of macro and micro-nutrients (stimulation of optimal body growth and development), **(C)** Active diet therapy (stimulation of immune tolerance); and the oral immunotherapy in combination with biologics in case of food allergy persistence.

Some negative factors influencing GM structure and function in early life (e.g., caesarean delivery, antibiotics and proton pump inhibitors use, UPF consumption, and anti-septic agents exposure) lead to a decrease in microbial diversity, and to a dysfunctional GM, resulting in lower production of immunomodulatory compounds (i.e., such as short chain fatty acids butyrate) playing a crucial role in regulating immune tolerance mechanisms and in the differentiation of regulatory T (Treg) cells (Carucci et al., 9, 10) (Figure 1).

## Food allergy prevention

The breakthrough of this concept has led to a paradigm shift in FA prevention. Indeed, for many years, allergen avoidance was the mainstay of FA prevention in infants at risk; but more recent evidence suggests that delaying the introduction of allergenic foods not prevent sensitization and could even increase the risk of developing FA, as described by Nuyttens et al. in this research topic. Accordingly, the most recent EAACI recommendations for FA prevention, emphasize the protective role of early introduction of allergenic foods in infants at high risk for food allergies (12). In addition, avoiding the use of drugs (i.e., antibiotics, proton pump inhibitors), promoting breastfeeding, and a diet rich in fibres, fermented foods, antioxidants, and omega-3 for the mother and their babies, could be effective strategies for FA prevention (Carucci et al., 9) (Figure 1).

## Dietary management and treatment approaches

In the interesting review provided by Leone et al., the authors described how the dietary management of pediatric gastrointestinal FA has been only based on strict avoidance of identified allergens, for years. For cow's milk allergy (CMA) management, in addition to CM avoidance, the use of substitutes formula is the mainstay of CMA treatment, with the use of different formulae based on the severity of FA, as underlined in the review paper of Robert E. et al., comparing the opinions of a group of Middle East expert with those of the European Society of Paediatric Gastroenterology, Hepatology and Nutrition (ESPGHAN) experts, in CMA symptoms and management (Robert et al.). During the last decades, the introduction of the “GM immune system axis” concept changed the FA management, passing from a passive to a pro-active approach (9).

The modern dietary management of pediatric FA requires a personalized approach aimed not only to the education about allergen avoidance, and to ensure an optimal growth and development; but it also includes an active diet therapy approach, exploiting the ability of some foods to positively modulate the “GM-immune system axis” potentially facilitating the occurrence of immune tolerance (9, Leone et al.).

Lastly, in the review of Leone et al., other therapies, such as the oral immunotherapy (OIT), are described, offering new hope by gradually desensitizing children through controlled food antigen exposure, with the help of biological therapies to reduce potential side effects and to increase the therapeutic efficacy of OIT (Leone et al.).

## Future directions: addressing the rising burden

The spread of pediatric gastrointestinal FA urgently requires comprehensive public health strategies for the prevention, and management of these condition.

Clarifying how, why, and when the FA prevention should start, and highlighting the importance of an integrated active approach able to positively modulate the “GM-immune system axis”, may significantly impact the disease course of pediatric gastrointestinal FA. The four articles of this research topic, cover all aspect of pediatric gastrointestinal FA starting from the pivotal role of immune dysregulation mechanisms and environmental factors in FA pathogenesis; and ending with the most recent evidence for a modern management of pediatric FA (Figure 1).
